# Letter from the Editor-in-Chief

**DOI:** 10.19102/icrm.2017.080106

**Published:** 2017-01-15

**Authors:** Moussa Mansour


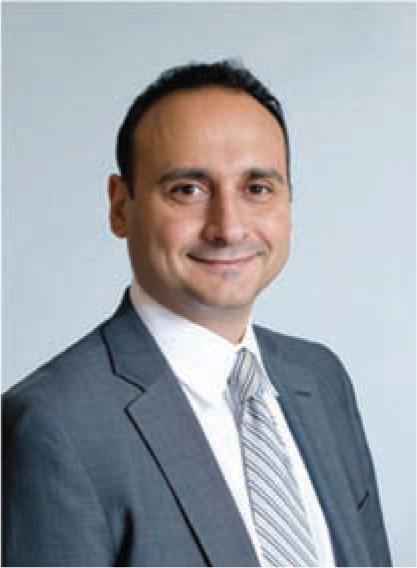


Dear Readers,

As of recently, the field of ambulatory monitoring for atrial fibrillation (AF) has been rapidly expanding. This explosive growth has been driven by several factors, including improvements made in monitoring devices; an increased number of AF ablation procedures; and the release of landmark studies demonstrating the role of AF in cryptogenic stroke. This issue of the *Journal* contains an important review article titled “Monitoring for AF: Identifying the Burden of Atrial Fibrillation and Assessing Post-Ablation.” In this publication, Dr. Rod Passman elegantly outlines the limitations of the conventional definition of clinically-relevant AF duration, and makes the case for the need for wide-scale long-term monitoring that will allow for the personalization of anticoagulation in AF patients, and possibly lead to safer and more cost-effective care.

I would like to highlight one area discussed within the article, which is post-AF ablation monitoring. This is a critically important topic because it is linked to post-procedure anticoagulation. While the use of oral anticoagulation prior to the ablation procedure is well-defined and agreed-upon, the optimal duration of anticoagulation following ablation is still under consideration. There are significant variations in the practice of rhythm monitoring and in prescribing anticoagulation after the procedure. At one end of the spectrum, patients are continued on oral anticoagulation, regardless of the outcome of the procedure, while on the other end, anticoagulation is stopped based on the elimination of AF symptoms determined without extensive monitoring. There are safety concerns with regards to both of these extreme practices: the continuation of anticoagulation in patients after successful ablation may lead to unnecessary bleeding, while stopping the medication in patients with continuing asymptomatic AF may lead to stroke. As a result, I believe that there is a need for extensive monitoring after ablation, which can help to better guide anticoagulation practices following ablation. This task has been made easier with the availability of new generation wearable and implantable devices that allow for long-term monitoring without significant discomfort to the patient.

In the United States alone, 75,000 to 100,000 AF ablations are performed every year; and this number is rapidly growing. Successfully monitoring this large number of patients for prolonged periods of time requires significant resources. Though the current monitoring devices contain many advanced features, processing the data and relaying the necessary information to the health care provider remains labor-intensive. As such, it is important that device manufacturers invest in the creation of improved signal-processing tools, as well as in technologies that allow for the seamless integration of the findings into the patient’s medical records.

Best regards, and I hope that you enjoy reading this issue of the *Journal*.

Sincerely,


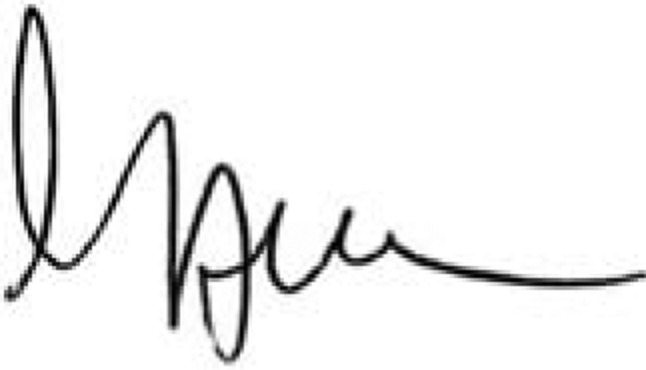


Moussa Mansour, MD, FHRS, FACC

Editor-in-Chief

The Journal of Innovations in Cardiac Rhythm Management

MMansour@InnovationsInCRM.com

Director, Cardiac Electrophysiology Laboratory

Director, Atrial Fibrillation Program

Massachusetts General Hospital

Boston, MA

